# Urinary arsenic species concentration in residents living near abandoned metal mines in South Korea

**DOI:** 10.1186/s40557-016-0150-z

**Published:** 2016-11-22

**Authors:** Jin-Yong Chung, Byoung-Gwon Kim, Byung-Kook Lee, Jai-Dong Moon, Joon Sakong, Man Joong Jeon, Jung-Duck Park, Byung-Sun Choi, Nam-Soo Kim, Seung-Do Yu, Jung-Wook Seo, Byeong-Jin Ye, Hyoun-Ju Lim, Young-Seoub Hong

**Affiliations:** 1 Heavy Metal Exposure Environmental Health Center, Dong-A University, Busan, Korea; 2 Department of Preventive Medicine, College of Medicine, Dong-A University, 26, Daesingongwon-ro, Seo-gu, Busan, Korea; 3 Korean Industrial Health Association, Seoul, Korea; 4 Department of Preventive and Occupational Medicine, Chonnam National University Hwasun Hospital, Hwasun, Korea; 5 Department of Preventive Medicine, College of Medicine, Yeungnam University, Daegu, Korea; 6 Department of Preventive Medicine, College of Medicine, Chung-Ang University, Seoul, Korea; 7 Institute of Environmental and Occupational Medicine, College of Medicine, Soonchunhyang University, Asan, Chungnam Korea; 8 National Institute of Environmental Research, Incheon, Korea; 9 Department of Occupational and Environmental Medicine, Dong-A University Hospital, Busan, Korea

**Keywords:** Abandoned metal mine, Arsenic, Arsenic species

## Abstract

**Background:**

Arsenic is a carcinogenic heavy metal that has a species-dependent health effects and abandoned metal mines are a source of significant arsenic exposure. Therefore, the aims of this study were to analyze urinary arsenic species and their concentration in residents living near abandoned metal mines and to monitor the environmental health effects of abandoned metal mines in Korea.

**Methods:**

This study was performed in 2014 to assess urinary arsenic excretion patterns of residents living near abandoned metal mines in South Korea. Demographic data such as gender, age, mine working history, period of residency, dietary patterns, smoking and alcohol use, and type of potable water consumed were obtaining using a questionnaire. Informed consent was also obtained from all study subjects (*n* = 119). Urinary arsenic species were quantified using high performance liquid chromatography (HPLC) and inductively coupled plasma mass spectrometry (ICP/MS).

**Results:**

The geometric mean of urinary arsenic (sum of dimethylarsinic acid, monomethylarsonic acid, As^3+^, and As^5+^) concentration was determined to be 131.98 μg/L (geometric mean; 95% CI, 116.72–149.23) while urinary inorganic arsenic (As^3+^ and As^5+^) concentration was 0.81 μg/L (95% CI, 0.53–1.23). 66.3% (*n* = 79) and 21.8% (*n* = 26) of these samples exceeded ATSDR reference values for urinary arsenic (>100 μg/L) and inorganic arsenic (>10 μg/L), respectively. Mean urinary arsenic concentrations (geometric mean, GM) were higher in women then in men, and increased with age. Of the five regions evaluated, while four regions had inorganic arsenic concentrations less than 0.40 μg/L, one region showed a significantly higher concentration (GM 15.48 μg/L; 95% CI, 7.51–31.91) which investigates further studies to identify etiological factors.

**Conclusion:**

We propose that the observed elevation in urinary arsenic concentration in residents living near abandoned metal mines may be due to environmental contamination from the abandoned metal mine.

**Trial registration:**

Not Applicable (We do not have health care intervention on human participants).

## Background

Arsenic is a naturally occurring element that is widely found in ground water and agricultural products and is one of the most abundant elements in the earth’s crust. Chronic arsenic exposure in humans is associated with diseases such as skin, lung, and hepatic cancer [1–3] and the primary sources of human exposure include ingestion of inorganic arsenic from contaminated water [4], industrial waste, pesticides, and inadequate mine waste disposal [5–7]. As most arsenic metabolites are soluble in water, they contaminate river and underground water, and arsenic contamination in ground water is categorized as a serious public health hazard. According to the United States Agency for Toxic Substances and Disease Registry (ATSDR) [8], the oral route is considered to be a predominant means of arsenic exposure in the general population. Several studies have also reported that exposure to or consumption of, even low levels, arsenic can lead to carcinogenesis [9–11]. In the last few decades, many studies have measured urinary arsenic concentration (organic and inorganic arsenic) and many older studies have used urinary arsenic as a biomarker of recent arsenic exposure. This latter approach, however, became obsolete as certain foods also contain organic arsenic which is similarly excreted in urine. As each arsenic species has different physiological and bioactive properties, separation of urinary arsenic metabolites is considered sufficient to both prevent potential overestimation of arsenic concentration and assess health risk [12, 13].

Metal mining was economically important in during the 19^th^ century, however, many mines were abandoned because of the change in industrial and economic conditions during the late 1970s [14]. Such abandoned metal mines have been identified as an important source of environmental heavy metal contamination and elevated levels of these toxic elements are often present in the soil and ground water of various countries [15, 16]. In many areas of Korea, there is evidence that the uncontrolled abandoning of these metal mines has had a large and lasting impact [17] as metals and metalloids dissolved from these mines may have contaminated both surface and ground water through solubilization into the surrounding environment. Further, as preventive measures to avoid environmental pollution after closures were not adequately implemented in some of these mines, debris from them, such as spoil heaps and water, remain potential source of environmental contamination. Therefore, this study was evaluated concentrations of urinary arsenic species in residents living near abandoned metal mines in Korea.

## Methods

### Study subjects and questionnaire

**Fig. 1:**
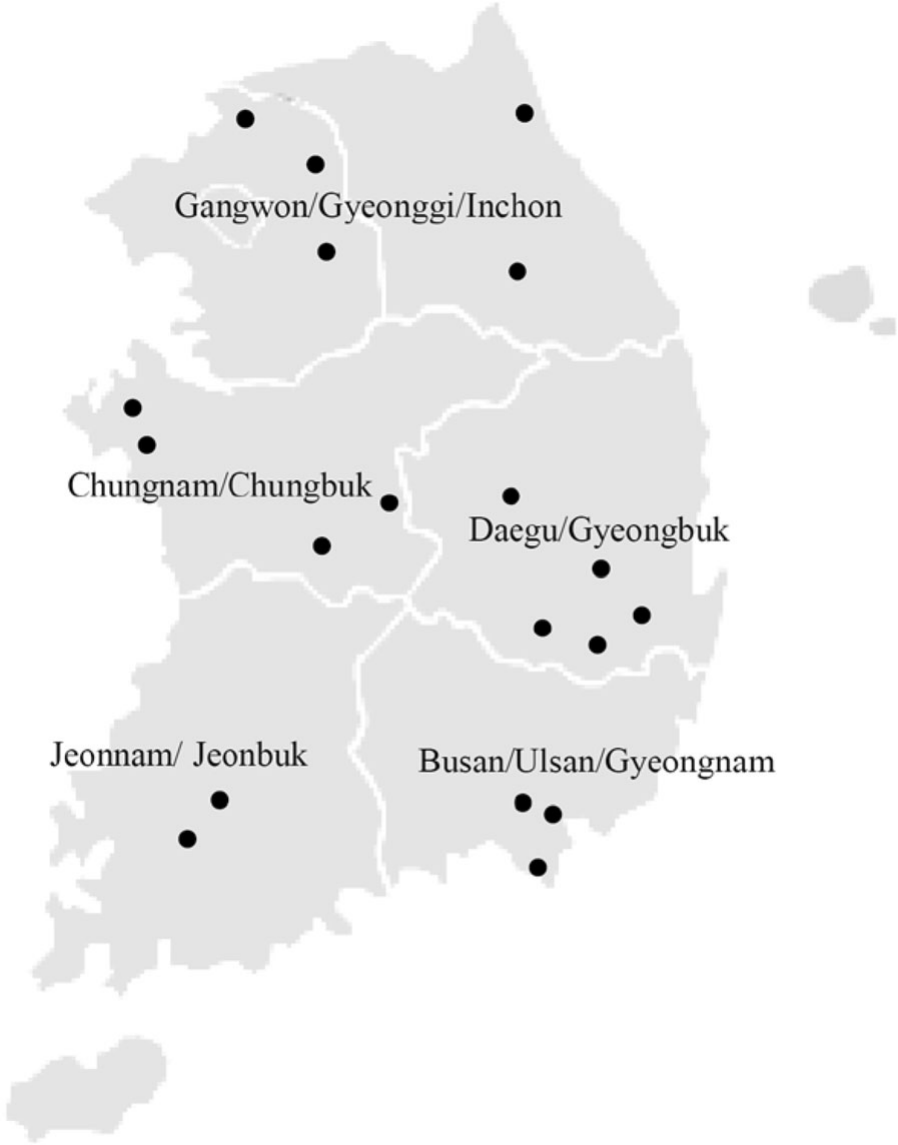
The five provinces and locations of abandoned metal mines in this study

Initially, we selected villages located within 3 km from the abandoned metal mines and the mine is located upstream of the each villages. urinary arsenic was measured in 974 samples obtained from residents living near abandoned metal mines using hydride generation-graphite furnace atomic absorption spectrometry (GFAAS). Subsequently, arsenic species analyses were carried out in a subset of samples with urinary arsenic concentrations in the 90^th^ percentile. Therefore this study analyzed urinary arsenic concentrations in 119 adults (45.4% male, 54.5% female) from residing near abandoned metal mines identified by the Ministry of Environment, Korea. The study included 19 villages located in East, West, South and Central Korea and was carried out between May and November, 2014. The abandoned mines are located in the Gangwon/Gyeonggi/Inchon (five villages), Daegu/Gyeongbuk (five villages), Busan/Ulsan/Gyeongnam (three villages), Jeonnam/Jeonbuk (three villages), Chungnam/Chungbuk (3 villages) regions of the Korean Peninsula (Fig. 1). These villages were selected as they are the most densely populated and are located within 3 km from the abandoned metal mines. Furthermore, the National Institute Environmental Research (NIER), Korea has conducted previous studies on heavy metals in farmland soil and drainage at abandoned metal mines area. The study was approved by the Institutional Review Board of the Dong-A University (ref. no. 2-1040709-AB-N-01-201404-BR-04-04). Informed consent was obtained from all participants and personal interviews were conducted to acquire demographic and lifestyle information such as age, drinking water source, current dietary habits, ongoing or previous disease, alcohol consumption, smoking status, type of drinking water being used and period of residency in the study area. Any history of working in mines was also obtained.

### Urine sample collection

All spot urine samples for organic and inorganic arsenic measurements were collected in disposable urine collection cups, placed in 15 ml polyethylene tubes, maintained at 4 °C, and transported to a laboratory where they were stored at – 70 °C till further analysis.

### Urinary arsenic speciation

Urinary arsenic species were analyzed at the Environmental Health Center, Dong-A university. Arsenic species were separated using an inductively coupled plasma mass spectrometry (ICP-MS) instrument (Agilent Technologies, Santa Clara, CA, USA) equipped with a high performance liquid chromatography (HPLC) system (Agilent 1260 Infinity, Agilent Technologies, Santa Clara, CA, USA). This methodology analyses As^3+^, As^5+^, monomethylarsonic acid (MMA), and dimethylarsinic acid (DMA) concentrations and the respective limits of detection are 0.17, 0.13, 0.19, and 0.12 μg/L. Urinary arsenic concentration was defined sum of the As3+, As5+, MMA, and DMA concentrations in this study. All urine samples were filtered through a 0.22 μm membrane before being placed into chromatographic vials, and the auto-sampler tray. Five-point calibration curves for all arsenic species tested (As^3+^, As^5+^, MMA and DMA) showed satisfactory linearity and the four major arsenic species could be separated within 16 min. The proportion of each arsenic species was calculated by dividing the concentration of that species by urinary arsenic. Replicate analyses of the standard reference material (SRM 2669, NIST standard, quality control) showed coefficient of variation to be less than 8%. For external quality assurance, we also completed both the occupational-medical and environmental-medical programs of German External Quality Assessment Scheme (G-EQUAS) of the Friedrich Alexander University, Erlangen.

### Statistical analysis

All statistical analyses were performed using the SAS statistical software (Version 9.4, SAS Institute, Cary, NC). Chi-square test for independence was performed for gender, age, period of residence, smoking, alcohol, type of drinking water, and history of working in mines. Arsenic species concentration is presented as unadjusted and adjusted geometric means with 95% confidence interval due to its right skewed distribution. The ANOVA and *t*-test were used to examine the relationship between demographic characteristics and arsenic species concentration. A *p*-value < 0.05 was considered statistically significant.

## Results

### Characteristics of the study population

The demographic characteristics of the study population are given in Table 1. The average age of the participants was 70.76 ± 9.17 years, and 58% of the participants were aged 70 or above. Among the 119 subjects, 14 subjects (11.8%) were smokers, 17 subjects (14.4%) reported previous history of working in mines, and 64 subjects (68.1%) used underground sources of potable water. Mean period of residence was 50.28 ± 23.77 years and was further categorized into three groups as ≤40, 41–60, and ≥61 years.

**Table 1:** General characteristics, including demographic and lifestyle data, of the study subjects

Characteristics		Total	Province					*p*-value
			Gangwon/Kyounggi/Inchon	Daegu/Gyeongbuk	Busan/Ulsan/Gyeongnam	Jeonnam/Jeonbuk	Chungnam/Chungbuk	
Total		119	25(21.0)	25(21.0)	14(11.8)	36(30.3)	19(16.0)	
Gender	Male	54(45.4)	7(28.0)	13(52.0)	4(28.6)	19(52.8)	11(57.9)	0.132
	Female	65(54.6)	18(72.0)	12(48.0)	10(71.4)	17(47.2)	8(42.1)	
Age(yr)	mean ± std	70.76 ± 9.17	71.44 ± 9.51	71.12 ± 7.10	70.57 ± 7.94	68.89 ± 11.01	73.11 ± 8.31	0.578
	41–59	13(10.9)	2(8.0)	1(4.0)	2(14.3)	7(19.4)	1(5.3)	0.541
	60–69	37(31.1)	8(32.0)	7(28.0)	3(21.4)	13(36.1)	6(31.6)	
	≥70	69(58.0)	15(60.0)	17(68.0)	9(64.3)	16(44.4)	12(63.2)	
Period fo residence(yr)	mean ± std	50.28 ± 23.77	40.04 ± 24.30	57.68 ± 21.56	52.93 ± 20.53	51.92 ± 25.41	48.42 ± 22.50	0.117
	≤40	35(29.7)	12(50.0)	3(12.0)	4(28.6)	11(30.6)	5(26.3)	0.178
	41–60	37(31.4)	7(29.2)	9(36.0)	3(21.4)	10(27.8)	8(42.1)	
	≥61	46(39.0)	5(20.8)	13(52.0)	7(50.0)	15(41.7)	6(31.6)	
Smoking	Current smoker	14(11.8)	2(8.0)	5(20.0)	2(14.3)	5(13.9)	0 (0.0)	0.379
	Ex-smoker	22(18.5)	3(12.0)	6(24.0)	2(14.3)	5(13.9)	6(31.6)	
	Non-smoker	83(69.7)	20(80.0)	14(56.0)	10(71.4)	26(72.2)	13(68.4)	
Alcohol	Drinking	64(53.8)	10(40.0)	18(72.0)	5(35.7)	23(63.9)	8(42.1)	0.048
	Non-drinking	55(46.2)	15(60.0)	7(28.0)	9(64.3)	13(36.1)	11(57.9)	
House income(KRW)	Below 500,000	63(52.9)	19(76.0)	11(44.0)	7(50.0)	18(50.0)	8(42.1)	0.035
	500,000–1,000,000	20(16.8)	1(4.0)	3(12.0)	3(21.4)	11(30.6)	2(10.5)	
	1,000,000–2,000,000	36(30.3)	5(20.0)	11(44.0)	4(28.6)	7(19.4)	9(47.4)	
Mine working history	Yes	17(14.4)	0 (0.0)	10(40.0)	1(7.1)	5(13.9)	1(5.6)	0.001
	No	101(85.6)	25(100.0)	15(60.0)	13(92.9)	31(86.1)	17(94.4)	
Dietary water	Tap water	30(31.9)	3(12.5)	3(50.0)	5(41.7)	10(27.8)	9(56.3)	0.037
	Underground water	64(68.1)	21(87.5)	3(50.0)	7(58.3)	26(72.2)	7(43.8)	
Herbicide	Yes	73(70.2)	13(52.0)	9(90.0)	10(71.4)	26(72.2)	15(78.9)	0.158
	No	31(29.8)	12(48.0)	1(10.0)	4(28.6)	10(27.8)	4(21.1)	

### Concentration of urinary arsenic species

Data on urinary arsenic species concentration in samples obtained from the five administrative provinces, as mean ± SD and GM with 95% CI, are given in Table 2. DMA was the predominant arsenic species (87.4%) in samples from all provinces, while MMA contributed to 6.5% of the urinary arsenic content. Mean (GM) urinary arsenic concentration, of all subjects, given as sum of As^3+^, As^5+^, MMA and DMA concentrations, was estimated to be 131.98 μg/L (95% CI, 116.72–149.23 μg/L). Samples from the Jeonnam/Jeonbuk province showed significantly higher urinary arsenic concentration (156.06 μg/L, 95% CI 114.28–213.12 μg/L) than those from other provinces. The concentration of inorganic arsenic species (As^3+^ and As^5+^) was significantly higher in samples from the Jeonnam/Jeonbuk province, however, levels of organic arsenic (DMA and MMA) were similar among samples from all the provinces. DMA levels contributed to 90–95% of urinary arsenic concentration in all samples except those from the Jeonnam/Jeonbuk province where it was 69%. Data on urinary arsenic species in relation to social-demographic variables are presented in Table 3. Even though urinary arsenic concentrations were higher in women than in men, the difference was not statistically significant, however, DMA concentrations were significantly higher in women than in men (*P* = 0.04). DMA concentrations was also higher in subjects with a history of working in mines compared to that in those without, but this difference was not statistically significant (*P* = 0.709). In case of period of residence, MMA concentration, same as As^3+^ concentration, showed significantly higher (*P* = 0.025) with increasing period of residence. Physiologically, As^3+^ methylated to form MMA which is further methylated to DMA [18]. Table 4 gives multivariate-adjusted geometric mean values for urinary arsenic concentration for each province, and urinary arsenic concentrations were similar among the province after adjustment. Inorganic arsenic levels exceeded the maximum reference levels in 8% of the samples from the Gangwon/Kyounggi/Inchon province and in 66.7% of the samples from the Jeonnam/Jeonbuk province (Table 5).

**Table 2:** Distribution of urinary arsenic species concentration (μg/L) in the populations living near abandoned metal mines at each province

Province	Number	Arsenic species		GM(95% CI), μg/L
Total	119	Organic	DMA	116.11(102.03–132.13)
			MMA	2.08(1.41–3.08)
		Inorganic	As^3+^	0.30(0.20–0.47)
			As^5+^	0.37(0.24–0.56)
		uAs		131.98(116.72–149.23)
Gangwon/Kyounggi/Inchon	25	Organic	DMA	138.69(112.83–170.47)
			MMA	0.73(0.35–1.52)
		Inorganic	As^3+^	<LOD(0.07–0.29)
			As^5+^	<LOD(0.07–0.26)
		uAs		144.39(116.80–178.51)
Daegu/Gyeongbuk	25	Organic	DMA	134.92(116.51–156.23)
			MMA	1.66(0.94–2.92)
		Inorganic	As^3+^	<LOD
			As^5+^	<LOD
		uAs		138.18(119.59–159.65)
Busan/Ulsan/Gyeongnam	14	Organic	DMA	117.29(89.51–153.71)
			MMA	0.73(0.25–2.12)
		Inorganic	As^3+^	<LOD
			As^5+^	<LOD
		uAs		119.88(91.22–157.53)
Jeonnam/Jeonbuk	36	Organic	DMA	108.36(76.45–153.59)
			MMA	22.61(17.47–29.26)
		Inorganic	As^3+^	4.96(2.31–10.61)
			As^5+^	7.10(4.26–11.82)
		uAs		156.06(114.28–213.12)
Chungnam/Chungbuk	19	Organic	DMA	85.33(62.65–116.22)
			MMA	0.26(0.14–0.50)
		Inorganic	As^3+^	<LOD
			As^5+^	<LOD
		uAs		86.25(63.42–117.30)

GM: geometric mean; CI: confidence interval

uAs: urinary arsenic(summation of As^3+^, As^5+^, MMA, and DMA)

As^3+^: trivalent arsenic or arsenite

As^5+^: pentavalent arsenic or arsenate

MMA: monomethylarsonic acid

DMA: dimethyarsinic acid

**Table 3:** Unadjusted geometric means of the arsenic species concentration in residents living near abandoned metal mines

Characteristics		Number	Organic As (μg/L)		Inorganic As (μg/L)		Urinary Arsenic (μg/L)
			DMA	MMA	As^3+^	As^5+^	
Total		119	116.11(102.03–132.13)	2.08(1.41–3.08)	0.30(0.20–0.47)	0.37(0.24–0.56)	131.98(116.72–149.23)
Gender	Male	54	100.55(83.55–121.02)	2.37(1.32–4.25)	0.36(0.19–0.69)	0.45(0.24–0.84)	118.42(100.40–139.68)
	Female	65	130.85(109.42-156.47)	1.87(1.09–3.20)	0.27(0.15–0.47)	0.31(0.18–0.55)	144.42(120.72–172.76)
	*p*-value		0.044	0.550	0.486	0.396	0.112
Age(yr)	41-59	13	95.17(61.56–147.12)	3.23(0.74–14.03)	0.54(0.11–2.73)	0.89(0.21–3.78)	116.28(76.34–177.12)
	60-69	37	102.00(80.18–129.78)	2.19(1.01–4.75)	0.46(0.19–1.08)	0.54(0.24–1.20)	121.18(98.10–149.67)
	≥70	69	129.21(109.33–152.70)	1.86(1.14–3.04)	0.22(0.13–0.37)	0.25(0.15–0.43)	141.50(119.71–167.26)
	*p*-value		0.150	0.694	0.202	0.088	0.415
Period of residence	≤40	35	104.43(83.68–130.32)	0.93(0.41–2.11)	0.26(0.12–0.56)	0.34(0.16–0.70)	117.03(94.82–144.43)
	41-60	37	139.65(111.45–174.99)	2.39(1.16–4.95)	0.25(0.11–0.56)	0.46(0.19–1.11)	156.52(123.50–198.37)
	≥61	46	107.73(85.53–135.70)	3.40(1.97–5.87)	0.41(0.20–0.85)	0.34(0.18–0.64)	125.50(102.29–153.98)
	*p*-value		0.154	0.025	0.581	0.781	0.159
Smoking	Current-smoker	14	79.08(44.38–140.91)	1.68(0.52–5.41)	0.20(0.06–0.65)	0.33(0.11–1.04)	97.16(64.31–146.79)
	Ex-smoker	22	118.72(94.31–149.46)	2.08(0.79–5.47)	0.33(0.11–0.99)	0.32(0.11–0.89)	133.37(103.50–171.87)
	Non-smoker	83	123.15(105.98–143.10)	2.16(1.34–3.48)	0.32(0.19–0.54)	0.39(0.23–0.65)	138.59(119.22–161.12)
	*p*-value		0.096	0.923	0.787	0.928	0.192
Alcohol	Drinking	64	102.89(85.73-123.48)	2.37(1.38-4.07)	0.34(0.19-0.60)	0.50(0.28-0.91)	121.14(103.12-142.31)
	Non-drinking	55	133.65(111.54-160.14)	1.79(1.00-3.20)	0.27(0.14-0.52)	0.26(0.14-0.45)	145.82(120.53-176.42)
	*p*-value		0.045	0.478	0.602	0.108	0.137
Income(KRW)	Below 500,000	63	116.52(96.59–140.55)	2.07(1.21–3.53)	0.28(0.16–0.50)	0.38(0.21–0.69)	131.29(109.08–158.02)
	500,000-1,000,000	20	106.50(71.20–159.29)	4.28(1.52–12.06)	0.58(0.17–2.00)	0.81(0.26–2.48)	131.59(95.09–182.10)
	1,000,000-2,000,000	36	121.07(100.01–146.55)	1.40(0.68–2.90)	0.25(0.11–0.54)	0.23(0.12–0.44)	133.42(110.09–161.70)
	*p*-value		0.813	0.180	0.389	0.137	0.993
Mine working history	Yes	17	124.94(92.57–168.63)	3.29(1.29–8.38)	0.32(0.09–1.10)	0.32(0.10–1.02)	139.92(105.61–185.38)
	No	101	116.66(101.27–134.38)	1.98(1.28–3.06)	0.31(0.19–0.49)	0.38(0.24–0.60)	133.09(116.44–152.11)
	*p*-value		0.709	0.370	0.928	0.792	0.773
Dietary water	Tap water	30	118.61(96.99–145.05)	1.72(0.72–4.10)	0.27(0.11–0.68)	0.55(0.22–1.37)	134.40(108.52–166.46)
	Underground water	64	112.52(91.42–138.48)	2.56(1.44–4.56)	0.56(0.30–1.05)	0.55(0.30–1.00)	133.40(110.23–161.45)
	*p*-value		0.713	0.438	0.203	0.996	0.962
Herbicide	Yes	73	111.02(93.53–131.78)	2.22(1.30–3.78)	0.34(0.19–0.61)	0.52(0.30–-0.93)	128.19(108.42–151.56)
	No	31	119.48(89.07–160.27)	1.70(0.75–3.81)	0.46(0.19–1.13)	0.33(0.15–0.72)	137.37(105.42–179.00)
	*p*-value		0.651	0.583	0.561	0.359	0.655

Urinary arsenic: summation of As^3+^, As^5+^, MMA, and DMA

**Table 4:** Adjusted geometric means for each province

		Adjusted GM(95% CI), μg/L						*p*-value
		Total	Gangwon/Kyounggi/Inchon	Daegu/Gyeongbuk	Busan/Ulsan/Gyeongnam	Jeonnam/Jeonbuk	Chungnam/Chungbuk	
oAs	DMA	92.04(63.09–134.28)	108.01(62.46–186.77)	106.67(54.03–210.59)	86.67(48.54–154.76)	98.25(64.67–149.28)	58.02(33.86–99.43)	0.161
	MMA	4.35(1.36–13.93)	1.22(0.48_-_3.11)^a^	0.45(0.14–1.44)^ac^	0.84(0.31–2.24)^ac^	32.80(16.08–66.89)^b^	0.24(0.09–0.59)^c^	<0.001
	Subtotal	106.62(74.49–152.62)	113.69(68.39–189.02)^a^	112.00(59.57–210.56)^a^	90.88(53.06–155.66)^a^	124.11(84.17–182.98)^a^	61.56(37.34–101.48)^a^	0.040
iAs	As^3+^	0.84(0.23–3.04)	0.14(0.04–0.46)^a^	0.13(0.03–0.56)^a^	0.07(0.02–0.23)^a^	6.18(2.50–15.32)^b^	0.06(0.02–0.20)^a^	<0.001
	As^5+^	0.83(0.24–2.87)	0.12(0.04–0.34)^a^	0.10(0.03–0.36)^a^	0.10(0.03–0.28)^a^	6.55(3.03–14.17)^b^	0.05(0.02–0.15)^a^	<0.001
	Subtotal	1.84(0.53–6.43)	0.30(0.11–0.76)^a^	0.24(0.07–0.77)^a^	0.15(0.06–0.41)^a^	15.48(7.51–31.91)^b^	0.11(0.04–0.27)^a^	<0.001
uAs		115.84(80.17–167.38)	116.59(70.11–193.87)^ac^	113.73(60.47–213.88)^ac^	91.91(53.64–157.46)^ac^	144.03(97.67–212.39)^b^	61.38(37.22–101.21)^c^	0.008

Adjusted: gender, age, period of residency, smoking, alcohol, house income, history of working in mines, dietary water, and herbicide

^abc^: according to regional differences, Scheffe’s post hoc grouping; the same letters are not significantly different

*oAs* organic arsenic, *iAs* inorganic arsenic, *uAs* urinary arsenic (summation of As^3+^, As^5+^, MMA, and DMA)

**Table 5:** The number of samples whose levels exceeded the reference levels of inorganic arsenic in each province

		Number	Number of exceed reference levels (%) Inorganic arsenic (>10 μg/L)^a^
Total		119	26(21.8)
Province	Gangwon/Kyounggi/Inchon	25	2(8.0)
	Daegu/Gyeongbuk	25	0(0.0)
	Busan/Ulsan/Gyeongnam	14	0(0.0)
	Jeonnam/Jeonbuk	36	24(66.7)
	Chungnam/Chungbuk	19	0(0.0)

^a^ATSDR (The American Agency for Toxic Substances and Disease Registry) Reference Levels

## Discussion

This study analyzed urinary arsenic species and their concentration in populations living near abandoned metal mines using HPLC-ICP-MS, and to the best of our knowledge, is the first to do so. The study population comprised only adult, and as participation was voluntary, the demographic data and results presented here are not representative of the general population. A spot urine sample was used for analyses because 24 h urine collection is uncomfortable and often results in improper or incomplete collection. Samples obtained from residents living near one abandoned metal mine located in the Jeonnam/Jeonbuk province alone showed significantly higher concentrations of urinary arsenic and inorganic arsenic. Moreover, life style and demographic status of residents in the Jeonnam/Jeonbuk province was not significantly different compare with other provinces. Potable water and food are important sources of human arsenic exposure, and urinary arsenic concentrations have been reported to be higher in populations living near contaminated areas compared to those residing in uncontaminated area [19–21]. Based on the health risks associated with arsenic exposure, the United States Environmental Protection Agency (USEPA) has established a reference level of 10 μg/L for dietary water [22]. According to the Survey of the Heavy Metals on Farmland Soil and Drainage at Abandoned Mine Area by the National Institute of Environmental Research in Korea (NIER, 2008), arsenic contamination of farmland soil and water within a distance of 2 km from an abandoned metal mine in the Jeonnam/Jeonbuk province exceeded both the preliminary standard and countermeasure standard for arsenic concentration. The Korean National Environmental Health Survey [23] has reported urinary arsenic concentrations of 35.0 μg/L (GM; 95% CI, 33.8–36.2 μg/L) in the general population aged over 20 years: using the hydride generation method. However, we show that mean urinary arsenic concentration (GM) in populations living near abandoned metal mines is significantly higher than that reported in the NIER survey. This discrepancy could be due to food consumption patterns, as food is an important source of organic arsenic (DMA and MMA) and a possible confounding factor during urinary arsenic species analysis. DMA is found in food as it is the end product of the arsenic metabolic pathway, irrespective of the arsenic species entering the living organism. In addition, seaweed and seafood contain arsenosugars that are converted to DMA after consumption and is excreted as such in urine [24]. Even though we recommended that all participants refrain from seaweed and seafood consumption for at least 3 days prior to sampling, it could not be strictly enforced. We observed that women had significantly higher DMA concentrations than men (*P* = 0.044), probably because available literature suggests that women can more efficiently methylate arsenic compared to men [25–27]. Our data show that DMA is the predominant arsenic metabolite in urine (69.2–95%) followed by MMA (1.2–14%), and inorganic arsenic (0.3–7.7%). Previous studies have reported similar results where DMA contributed to 84–86% of the urinary arsenic species [28, 29]. In 2000, a WHO report estimated that the arsenic content of cigarette smoke was 40–120 ng per cigarette [30]. However, we observed that smoking was not a significant determinant of urinary arsenic species concentration.

## Conclusion

We show that residents living near abandoned metal mines are not markedly overexposed to arsenic except in the case of one abandoned metal mine in the Jeonnam/Jeonbuk province. A probable reason for this observation is consumption of arsenic-contaminated ground water from near the abandoned metal mine.

A limitation of our study is that the arsenic exposed group did not have comparable control subjects. Further, only 10% of the 974 samples were subjected to arsenic species analyses. We, therefore, suggest that all future studies implement arsenic species analysis in all samples rather than only in a subset. We, meanwhile, could not survey the consumption of seafood and seaweed at that time. But, we are going to consider assessment the relationship between arsenic concentration and seafood consumption in the next study. As there are no reports on the analysis of urinary arsenic species using HPLC-ICP-MS in populations living near abandoned metal mines in Korea, this study provides valuable data on the prevalence and concentration of urinary arsenic species in arsenic exposed populations, especially abandoned metal mine area.
